# Supplementary figures and images for: Functional Identification and Regulatory Active Site Screening of the DfDXS Gene of Dryopteris fragrans
Source: Plants (Basel). 2024 Sep 21;13(18):2647. doi: 10.3390/plants13182647 (PMC11435244; doi:10.3390/plants13182647)

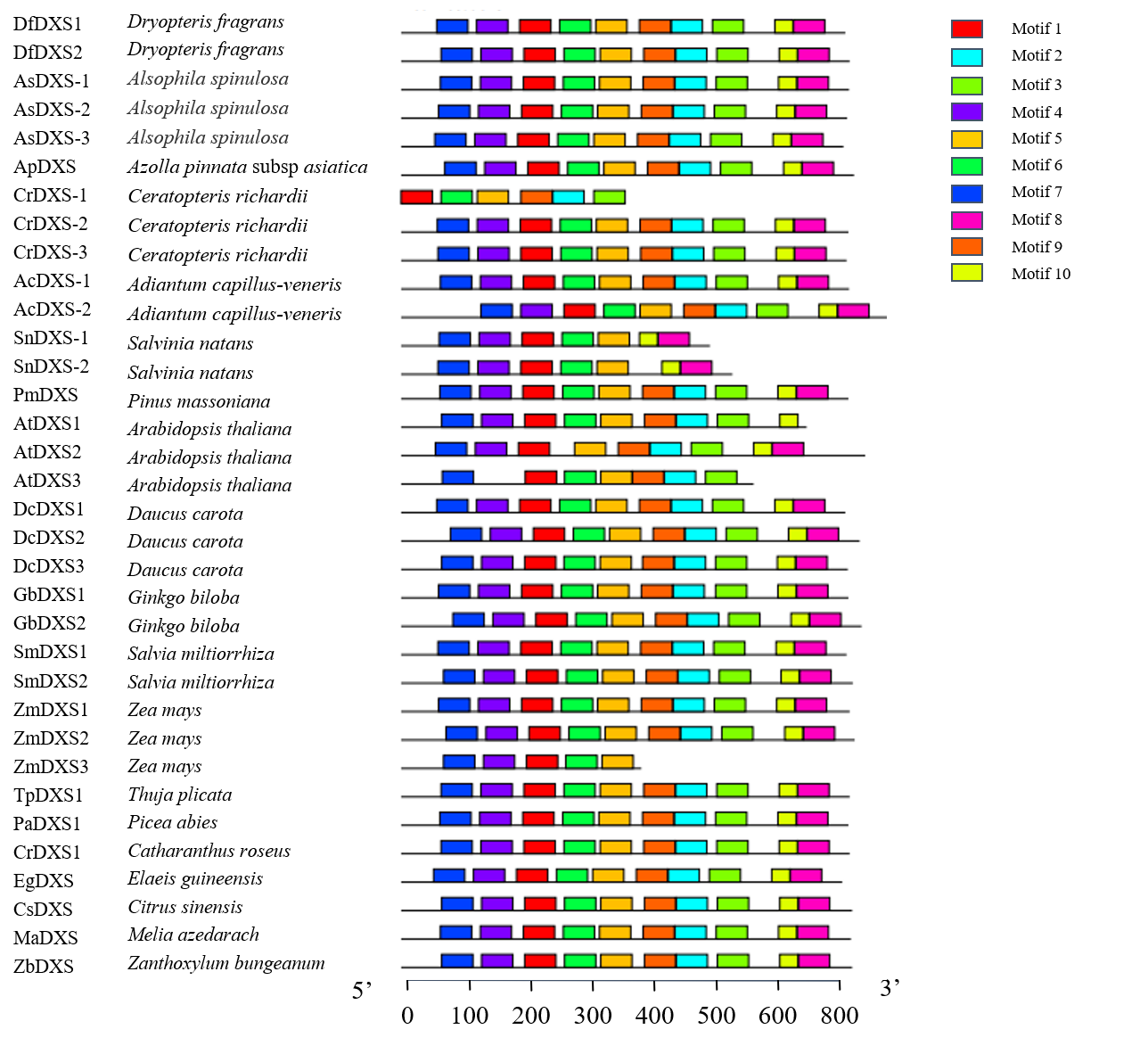

Supplement: Supplementary file 1 [file plants-13-02647-s001.zip › Figure S1.png]

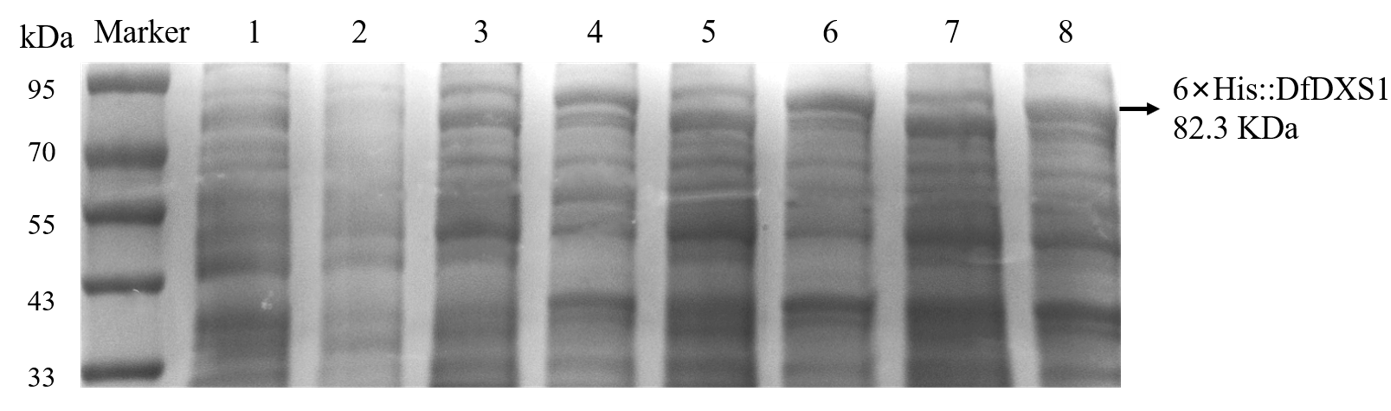

Supplement: Supplementary file 1 [file plants-13-02647-s001.zip › Figure S2.png]

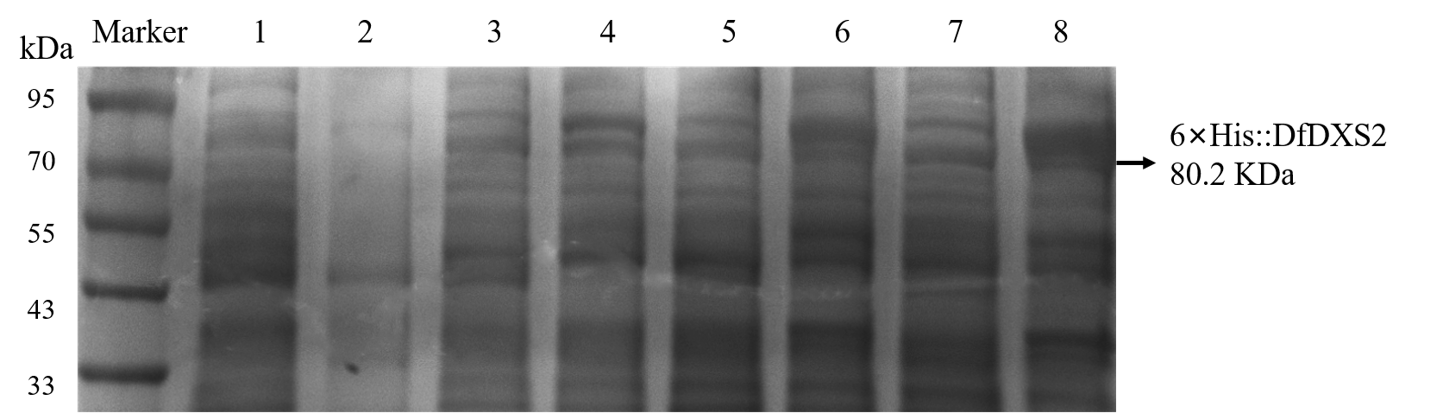

Supplement: Supplementary file 1 [file plants-13-02647-s001.zip › Figure S3.png]
